# The Effects of Language and Semantic Repetition on the Enactment Effect of Action Memory

**DOI:** 10.3389/fpsyg.2020.00515

**Published:** 2020-03-20

**Authors:** Xinyuan Zhang, Sascha Zuber

**Affiliations:** ^1^School of Psychology, Northeast Normal University, Changchun, China; ^2^Jilin Provincial Experimental Teaching Demonstration Centre of Psychology, Northeast Normal University, Changchun, China; ^3^Department of Psychology, University of Geneva, Geneva, Switzerland; ^4^Center for the Interdisciplinary Study of Gerontology and Vulnerability, University of Geneva, Geneva, Switzerland; ^5^Swiss National Centre of Competence in Research LIVES – Overcoming Vulnerability: Life Course Perspectives, Geneva, Switzerland

**Keywords:** enactment effect, action memory, semantic, language, multimodal theory

## Abstract

Humans exhibit enhanced memory performance when information is encoded by physically enacting it, as opposed to passively reading or hearing the same information; an effect referred to as “enactment effect.” The present study explored the effects of language (native vs. non-native) and semantic repetition (repeated vs. non-repeated) on the enactment effect in action memory. Forty-eight subjects learned action phrases either by enacting or by reading the items. Results showed (i) better memory for enacted phrases, (ii) better memory for non-native repeated phrases that were only read, (iii) no difference in memory between repeated and non-repeated phrases that were enacted, and (iv) that semantic repetition affected memory of phrases that were read but not of those that were enacted. Partly in line with the multimodal theory, findings support that enacting action phrases can enhance item-specific processing and that this is insensitive to cognitive strategies like semantic repetition.

## Introduction

For over 30 years, an active line of studies has investigated the enactment effect, a well-established effect indexed by enhanced memory performance for enacted learning compared to verbal learning (for a review, see Engelkamp, 1998; Nilsson, 2000). In a typical action memory study, subjects are asked to learn a set of action phrases consisting of a simple combination of verbs and objects like, “peel the banana,” or “click the mouse.” These action phrases can be learned in two main conditions: in the subject-performed task condition (SPT), subjects are required to read and enact the action phrases, whereas in the verbal task condition (VT) subjects have to read the phrases without enacting them. Subjects typically recall more phrases that they have also enacted compared to those they have only read. The enactment effect represents a robust phenomenon, as it has been observed in a large number of studies across various encoding-, recall-, and recognition-conditions (e.g., Engelkamp and Krumnacker, 1980; Cohen, 1981, 1989; Saltz and Donnenwerth-Nolan, 1981; Bäckman et al., 1986; Engelkamp and Zimmer, 1994; Engelkamp et al., 1994; Schatz et al., 2011; Steffens et al., 2015; Zhao et al., 2016; Badinlou et al., 2017, 2018a,b; Hainselin et al., 2017; Li et al., 2017; Wammes et al., 2017; Yu and Wang, 2017; Borg et al., 2018; Li and Wang, 2018; Liu and Wang, 2018). Interestingly, studies found that enactment can also enhance learning foreign languages (see also Macedonia and von Kriegstein, 2012) and can thereby facilitate the process of language acquisition (for reviews, see Taleghani-Nikazm, 2008).

Despite the fact that researchers in the field firmly acknowledge the enactment effect, they fail to agree on the mechanisms behind the phenomenon. On one side, the dual-code theory suggests that by reading or hearing a phrase it is only represented linguistically, but that by enacting a phrase, the corresponding representation is created both motorically and linguistically, which ultimately leads to the recall advantage (e.g., Engelkamp and Zimmer, 1985; Zimmer and Engelkamp, 1985; Cohen, 1989; Foley and Ratner, 2001; Zimmer et al., 2001). The multimodal theory can be seen as an extension of the dual-code theory (Zimmer et al., 2001; Knopf et al., 2005). It declares motor encoding as the key factor of the enactment effect: in addition to verbal encoding and visual encoding the motoric encoding would provide a fundamental advantage to memory performance after encoding by enactment, because it activates (and later reactivates) information stored in the motor system (Engelkamp and Zimmer, 1984; Zimmer et al., 2001; Ianì and Bucciarelli, 2018).

Although multimodal theory focus on the advantage of deploying the motor system, the specific role played by the motor system in the enactment effect is a topic of ongoing discussion (Helstrup, 1987, 2005; Engelkamp, 2001; Nilsson and Kormi-Nouri, 2001). Certain studies provide evidence against the assumption of a key role of the motor system in the enactment effect (see Kormi-Nouri and Nilsson, 2001; Knopf et al., 2005). Alternatively, opposing theories suggest that there is no motor system signal supporting the enactment effect. Instead, action memory would be facilitated by conceptual processes (Zimmer et al., 2001). More specifically, the episodic integration theory postulates that action memory is facilitated by integrating the action (e.g., “to peel”) and the item (e.g., “the banana”) into a single information unit or into two closely related units (Kormi-nouri, 1995; Kormi-Nouri and Nilsson, 1998, 2001; Feyereisen, 2009). In addition to item-specific information (e.g., the specific color and form and shape of a banana), enactment would also activate relational information (i.e., encoding similarities among a class of phrases). Further, enactment would improve memory performance because of the high level of self-involvement, promoting integration not only within and between items (i.e., previously acquired general knowledge of the relation between verbs and nouns or between action phrases), but also between the subject (i.e., the rememberer) and the environment (i.e., the task of having to enact the phrase; Kormi-nouri, 1995; Kormi-Nouri and Nilsson, 1998; Zimmer et al., 2001; Hainselin et al., 2014).

In view of these opposing theoretical models, how can one investigate the mechanisms that underlie the enactment effect? One possibility is to investigate different factors that may affect the enactment effect (Feyereisen, 2009). For that purpose, prior research has traditionally focused on the motor information (e.g., Engelkamp, 2001; Russ et al., 2003; von Essen and Nilsson, 2003; Knopf et al., 2005) or on the action representations of the enactment effect (see, e.g., Kormi-nouri, 1995; Masumoto et al., 2006; Spranger et al., 2008). However, a growing number of studies show that, beside the action representations and the motor information, different linguistic properties of the phrases that have to be learned can also influence the effect. For instance, a line of studies investigated the role of semantic integration, which designates the semantic association between verb and noun in the action phrases. Typically, in these studies, memory performance on well-integrated phrases like “read the book” (high semantic association between verb and noun) is compared with poorly integrated phrases like “push the cup” (low semantic association) in SPT or VT conditions (e.g., Badinlou et al., 2018a). Results show that in both types of phrases performance is higher in SPT compared to VT condition, but also that well-integrated phrases are better recalled in both VT and SPT condition (Kormi-nouri, 1995; Kormi-Nouri and Nilsson, 1998; Kormi-Nouri et al., 2003; Badinlou et al., 2018a, b).

Although this demonstrates that linguistic properties such as the semantic integration of an action phrase can influence the enactment effect, the influence of potential other linguistic properties has to be explored. For example, Eysenck and Keane (1995) have conceptualized how language is represented in memory. They postulate that language representations can be distinguished into external representations (e.g., different languages, words or sentences) and internal representations (e.g., semantic properties, concepts). Based on this distinction, the current study set out to investigate how two of these linguistic properties and their interaction will influence the enactment effect. Specifically, we aimed to investigate the effects of external and internal language representation on the enactment effect (i) by examining memory of phrases in native versus in non-native language, and (ii) by examining memory of semantically repeated versus non-repeated phrases.

In detail, regarding external language representations, the effectiveness of the paradigm makes it a great tool to investigate the beneficial effects of action memory in learning foreign languages (see also Macedonia and von Kriegstein, 2012). Generally, the findings from previous studies reveal that the use of gestures to encode new information facilitates and enhances the process of language acquisition (for a review see, Taleghani-Nikazm, 2008) as well as verbal memory (e.g., Zimmer et al., 2001). This effect was replicated in various languages such as German (Engelkamp and Krumnacker, 1980; Krönke et al., 2013), Italian (Cutica et al., 2014; Ianì and Bucciarelli, 2017; Ianì and Bucciarelli, 2018; Ianì et al., 2018), Chinese (Li et al., 2017; Liu and Wang, 2018), and English (Schwartz and Plass, 2014). However, the aforementioned studies focused on one language at a time and were not designed to test the effect of different languages in the same study. Therefore, the current study first will examine whether and how the enactment effect interacts with different linguistics modalities by comparing native versus non-native phrases in a single study. This is conceptually important, because it allows examining whether the enactment effect is biased when using native versus non-native languages in international studies. If language would affect the enactment effect, the reliability of previous research results would have to be interpreted cautiously, whereas this were less the case if there are no differences between native and non-native languages.

Our second goal concerns the effect of semantic repetition on the enactment effect of different languages. In studies examining memory and learning, “repetition” typically refers to treating a particular item two times in exactly the same modality (e.g., encoding the English phrase “open the door” two times during the learning phase). In contrast, with “semantic repetition” we designate treating an item and its semantic equivalent in a different modality (e.g., encoding the English phrase “open the door” as well as its Chinese translation “
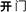
”). Two arguments suggest that semantic repetition could influence the enactment effect. On the one hand, the completion of an action requires semantic understanding, thus deeming semantic processing a prerequisite for action execution (Kausler and Hakami, 1983; Helstrup, 1989; Wippich and Mecklenbräuker, 1995; Steffens et al., 2003). For example, when reading the phrase “open the book,” subjects must first understand the meaning of this phrase and then perform the corresponding action. Studies show that compared to simply reading phrases when having to learn them, semantically “treating” information enhances memory (e.g., Zimmer and Engelkamp, 1999).

On the other hand, studies also show performance improvements following learning repetition. Studies investigating repeated learning in SPT showed improved memory performance, likely as a result of enhanced item-specific processing (Schatz et al., 2010, 2011; Kubik et al., 2014a, b). Thus, to combine the enhancing roles of deeper semantic treatment and repetition, the present study aimed to explore the effect of semantic repetition. If semantic repetition proved to enhance memory performance, this would importantly contribute to the previous literature as it suggests that it is not the mere repetition of an item, but that it is the deeper processing (i.e., the semantic processing) that has an enhancing effect.

Further, manipulating repetition may provide additional evidence to support or contradict the opposing theories explaining the mechanisms of the enactment effect. If the performance of SPT learning was not affected by semantic repetition, this would speak in favor of the multimodal theory, suggesting that additionally encoding phrases via the motor system would be the key factor leading to enhanced performance whereas other factors (such as semantic processing and repetition) would not contribute to the effect above and beyond motor encoding. In contrast, if the performance of SPT learning was affected by semantic repetition, this would speak in favor of the episodic integration theory, as it would suggest that beyond the enhancing effect of motorically encoding the items, semantically repeating these items would have an additive effect, by re-activating relational information between phrases in conceptual processing.

Finally, while internal representations (e.g., semantic processing) have been shown to interact with the enactment effect, there is no record of studies that combined the effect of semantic changes with external language properties. The present study will fill this gap in the literature by asking the question as to how the enactment effect is associated with the interplay between external and internal language representations, namely language (native vs. non-native) and semantic repetition (repeated vs. non-repeated).

Taken together, for the present study followed four research goals. First, we expected to replicate the enactment effect by finding superior memory performance for the SPT compared to the VT condition. Second, we aimed to examine whether there were differences in performance between the native versus non-native phrases. Third, we further expected that semantic repetition would lead to superior memory performance (compared to non-repeated phrases). Fourth, we aimed to examine potential interactions between language, semantic repetition, and enactment, which would provide further insight into the different theories explaining the mechanisms of the enactment effect.

## Materials and Methods

### Participants

Based on previous studies (e.g., Zimmer and Engelkamp, 1999), a power analysis performed using G^∗^Power revealed a minimal sample size of *N* = 44. To allow for potential exclusions, we recruited 48 undergraduate students from Northeast Normal University to take party in the current study. All 48 participants (27 males and 21 females; age: *M* = 23.46 years, *SD* = 1.53) met inclusion criteria and thus were included in subsequent analyses. Each participant received 15 Yuan RMB (∼2.30 USD) for their participation. All participants spoke Chinese as their native language and were proficient users of English as their non-native language (∼13 years of practice). None of the participants had previous experience in the current (or similar) experiment. All participants had normal or corrected-to-normal vision and had no history of neurological, psychological, or any other chronic illnesses. All participants gave written informed consent prior to their participation.

### Design

The present study adopted a 2 × 2 × 2 mixed design, with the type of encoding (SPT vs. VT) as between-subject factor, and with language (native vs. non-native) and semantic repetition (repeated vs. non-repeated) as within-subject factors. In the SPT condition, participants were required to read and to perform 48 action phrases, whereas, in the VT condition participants only had to read these phrases. In both conditions, subjects learned half (24) of the phrases in their native language (i.e., Chinese) and half of the phrases in a non-native language (i.e., English). For the manipulation of semantic repetition, 12 phrases in each language were cross-matched with their identical semantic counterpart in the other language (semantic repetition), for example, the phrase “open the door” in English was matched with its Chinese semantic equivalent “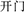.” The remaining 12 phrases in each language had unique semantics meanings that did not match across language (non-repetition). All phrases were presented in random order.

### Material

We used a total of 24 Chinese phrases and 24 English phrases. The Chinese phrases consisted of 2–4 Chinese characters, whereas the English phrases consisted of 3–4 words. Note that, the English phrases selected for the purpose of the study were easy to understand (i.e., open the door, click the mouse, or eat the apple.). To select these phrases, we asked 12 Chinese students of English as a foreign language to translate 100 phrases. Six students translated Chinese phrases to English, and six students translated English phrases to Chinese (the translation students did not participate in the formal experiment). At the end of this exercise, two groups of phrases with identical English-Chinese translations were selected to be used in the experiment. The recognition test included 96 action phrases, of which 48 had already been seen by participants during the experiment. The remaining 48 phrases were completely new and were used as interference stimuli in the recognition test. Half of the phrases in the recognition test were presented in Chinese, and the other half presented in English.

### Procedure

The experiment was programmed in E-prime 2.0 and was presented on a computer screen. All participants were instructed to learn and memorize the action phrases either by reading and enacting (SPT condition) or by only reading the action phrases (VT condition). Once participants understood the instructions, they started the learning phase. Each trial in the learning phase consisted of a randomly selected phrase that was presented for 8 s. Note that each phrase was only presented once per language (but could be repeated in the other language for the semantic repetition phrases). The 12 phrases that were semantically repeated were counterbalanced so that six phrases were first presented in English and six first in Chinese. At the end of the learning phase, the participants were given a 5-min distraction task which required them to solve a series of mathematical problems. Then, participants took part in a recognition task, where they responded to whether they recognized any of the given phrases as being previously presented in the learning phase. If a phrase was recognized, the participant had to press the “F” keyboard key. If they felt they had never seen the phrase during the experiment they had to press the keyboard key “J.” Following each keypress, the next phrase was presented. The participants were asked to respond as fast and as accurately as possible. The experiment lasted approximately 15 min (learning phase = 48 trials, ∼5 min; distraction task = ∼5 min; recognition task = 96 trials, ∼5 min).

### Memory Scores

The recognition test consisted of a discrimination task, where participants had to respond whether they recognized a given phrase as belonging to the experimental session, or if the phrase was being presented for the first time. This method was selected to avoid any score difference caused by spelling errors in case the participants had to perform free recall or follow cue recall. The recognition scores were analyzed using the d-prime (*d’*) measure, a parametric measure sensitive to item discrimination (see formula below). In this equation, H represents the hit rate (the proportion of old items participants correctly identified as “old”), and FA represents the false alarm rate (the proportion of new actions participants incorrectly identified as “old”).

$$d^{\prime }=z\,\left(H\right)-z\,\left(F\,A\right)$$

Three *d’* scores were calculated using separated hit rates based on the proportion of Chinese semantic-repeated phrases correctly recognized, the proportion of English semantic-repeated phrases correctly recognized, the proportion of Chinese semantic non-repeated phrases correctly recognized, and the proportion of English semantic non-repeated phrases correctly recognized. A single false alarm rate was used to calculate all four *d’* scores since false alarm rates were derived from performance on distractor items, which by definition did not belong to any of the categories aforementioned.

## Results

**FIGURE 1:**
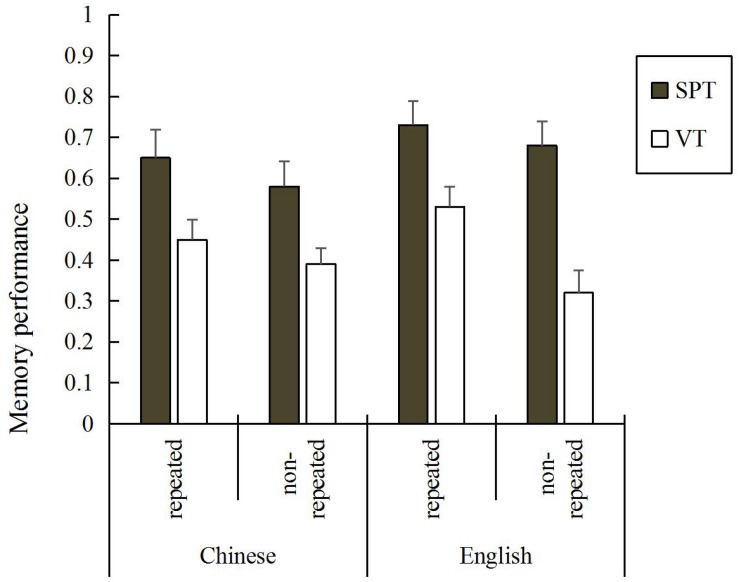
Mean memory performance per encoding condition, language, and semantic repetition condition. Bars represent standard errors.

Figure 1 depicts means and standard errors of memory performance, separated per condition, language and semantic repetition. To analyze the effects of encoding type, language and the semantic repetition on memory performance, a 2 (type of encoding: SPT vs. VT) × 2 (language: Chinese vs. English) × 2 (semantic repetition: repeated vs. non-repeated) mixed analysis of variance (ANOVA) was conducted. The ANOVA showed that the three factors had a significant effect on memory scores; encoding type, *F*(1,46) = 11.43, *p* = 0.001, η^2^ = 0.199, language, *F*(1,46) = 4.73, *p* = 0.035, η^2^ = 0.093, and semantic repetition, *F*(1,46) = 21.55, *p* < 0.001, η^2^ = 0.319. The ANOVA also revealed a significant three-way interaction, *F*(1,46) = 9.98, *p* = 0.003, η^2^ = 0.178.

Further, to examine whether there were different interactions between language and semantic repetition in the SPT versus the VT condition, we separated the analyses by encoding type. As for the SPT condition, the two-way interaction between language and semantic repetition was not significant, *F*(1,23) = 0.40, *p* = 0.533, η^2^ = 0.017. As for the VT condition, the interaction between language and semantic repetition was significant, *F*(1,23) = 26.87, *p* < 0.001, η^2^ = 0.539. Looking at the VT condition separately, regarding semantic repetition, analyses of simple effects show that English phrases were better recognized if they were repeated (*M* = 0.53, *SD* = 0.25) compared to phrases that were not repeated (*M* = 0.32, *SD* = 0.27), *t*(23) = 6.57, *p* < 0.001, Cohen’s *d* = 0.807. In contrast, in Chinese there was no difference between repeated phrases (*M* = 0.45, *SD* = 0.24) and non-repeated phrases (*M* = 0.39, *SD* = 0.19), *t*(23) = 1.69, *p* = 0.105, Cohen’s *d* = 0.277. Regarding language, repeated phrases were better recognized in English (*M* = 0.53, *SD* = 0.25) than in Chinese (*M* = 0.45, *SD* = 0.24), *t*(23) = 2.05, *p* = 0.05, Cohen’s *d* = 0.326. In contrast for non-repeated phrases there was no difference between English (*M* = 0.32, *SD* = 0.27) and Chinese (*M* = 0.39, *SD* = 0.19), *t*(23) = 1.91, *p* = 0.069, Cohen’s *d* = 0.300.

In a further analysis, we calculated the size of the enactment effect (mean *d’* scores (SPT–VT)/VT comparison) for both language and semantic repetition. Regarding language, we found that the size of the enactment effect in English phrases (mean *d’* scores = 0.67) was not significantly larger than in Chinese phrases (mean *d’* scores = 0.48), *Z* = 1.35, *p* > 0.05 (hypothesis tests of the difference between two population proportions; Anderson et al., 2011), indicating that language did not affect the size of the enactment effect. As for semantic repetition, the results showed that the size of enactment effect in the semantic repetition condition (mean *d’* scores = 0.42) was smaller than in the non-repetition condition (mean *d’* scores = 0.79), *Z* = −2.61, *p* < 0.01, indicating that semantic repetition affects the size of the enactment effect, with a stronger effect in the non-repetition condition.

## Discussion

The current study investigated how language (native vs. non-native) and semantic repetition (repeated vs. non-repeated) interacted with the enactment effect. Specifically, we pursued four main research goals: (i) replicating the enactment effect (ii) examining differences of the enactment effect in native versus non-native languages, (iii) examining whether semantic repetition would enhance memory performance and would be associated with the size of the enactment effect, and (iv) examining potential interactions between language, semantic repetition, and enactment to gain further insights into whether the multimodal theory or the episodic integration theory is more suitable for explaining the enactment effect.

Regarding the first goal, results showed an enactment effect, which was reflected in superior memory scores for phrases learned in the SPT compared to the VT condition. The present study thereby replicates and further supports an extensive line of action memory studies, providing further evidence for a robust enactment effect (e.g., Engelkamp and Krumnacker, 1980; Cohen, 1981; Saltz and Donnenwerth-Nolan, 1981; Steffens et al., 2015; Zhao et al., 2016; Hainselin et al., 2017; Li et al., 2017; Wammes et al., 2017; Yu and Wang, 2017; Ianì and Bucciarelli, 2018; Li and Wang, 2018; Liu and Wang, 2018). Under this premise, our subsequent research goals targeted a better understanding of how enactment enhances memory.

Specifically, regarding our second research goal of comparing a native with a non-native language, the current study demonstrated that memory performance was better for English (non-native language) compared to Chinese phrases (native language). One may naturally expect superior memory performance in the native language. However, one possible explanation for this effect could be that to some degree, the English phrases were more novel or more “bizarre” compared to Chinese phrases. This may have made English phrases more remarkable and thus easier to remember. This view is supported by an earlier study in which participants’ memories of bizarre or novel items were better than those of non-bizarre, non-novel items (Einstein and McDaniel, 1987).

It further is important to highlight that language is not a critical determinant of the size of the effect, as the size of the effect was not significantly different between English and Chinese phrases (i.e., no difference in d’ scores). The evidence that the effect manifested in both languages suggests that the language chosen for the study is not critical for the effect to emerge. This supports the reliability of findings from international studies that used the local language to study the enactment effect. Thus, if studies continue using different languages that are familiar to the local participants, this should not bias the results when investigating the enactment effect.

Regarding our third goal, semantic repetition improved memory performance, but only in the VT condition, indicating that the deeper processing related to semantically repeating certain phrases enhanced memory performance for VT learning, which was not beneficial in SPT learning. Indeed, previous studies suggest that SPT memory exhibits less sensitivity to cognitive strategies (e.g., rehearsal or organizational strategies) than does VT memory (e.g., Engelkamp, 1996; Zimmer et al., 2001; Gollyhäring and Engelkamp, 2003; Von-Essen, 2005). Similarly, they suggest that conceptual elaboration (that is, elaborating a concept with one’s knowledge; for example, for an apple, that it grows on fruit trees and matures in autumn) commonly had an influence on the enactment effect by affecting memory performance under the VT condition, but no (or a smaller) influence under the SPT condition (Nilsson and Craik, 1990; Cohen and Bryant, 1991; Zimmer and Engelkamp, 1999). In this line, our findings also support that SPT learning is rather insensitive to cognitive strategies. Specifically, as semantic repetition re-activates relational information, our study supports the idea that enacting action phrases can enhance memory via item-specific processing rather than via relational processing (e.g., Zimmer and Engelkamp, 1985; Seiler and Engelkamp, 2003; Steffens et al., 2006; Steffens et al., 2009; Schatz et al., 2011; Li and Wang, 2018).

Further, semantic repetition was associated with the size of the enactment effect. Specifically, the size of the enactment effect was smaller in the repeated compared to the non-repeated condition. In the (SPT-VT)/VT comparison, VT memory scores of semantically repeated phrases increased, making the difference between the two conditions smaller. In contrast, memory performance in the non-repeated semantic condition did not change in the same way. As the SPT learning was not affected by semantic repetition, this result again provides evidence that enactment seems to be enhanced by item-specific processing without being affected by relational information.

Regarding our fourth study goal of investigating the interaction of language, semantic repetition, and enactment, our results demonstrate memory improvements when participants learned repeated phrases in a foreign language (English) in the VT condition but not in the SPT condition. In contrast, the same effect was not observed in phrases learned in the native language. That is, the effect of semantic repetition was comparable for Chinese phrases learned in both SPT and VT conditions. Although the present study design does not allow to fully disentangle the exact mechanisms behind this finding, one possible explanation is that the effect of semantic repetition was suppressed by processing habits (i.e., participants’ preferred strategy when learning or remembering) in the native language. Although preliminary at this point, these findings are important because they demonstrate that while foreign language learning is susceptible to the principles of conceptual elaboration (as observed in the VT condition), the same is not true for the SPT condition. More precisely, our findings thereby support the notion that conceptual elaboration does not improve SPT recall (see Nilsson and Craik, 1990; Zimmer and Engelkamp, 1999).

As mentioned earlier, three main theories attempt to explain the enactment effect: the dual-code theory, the multimodal theory, and the episodic integration theory. These three explanations of the SPT advantage share the assumption that enactment adds something to the processing of the verbal material that has to be memorized (Feyereisen, 2009). The problem is to identify what exactly is added. For example, in the multimodal theory, Engelkamp (2001) emphasized the role of the motor system as key factor in the enactment effect. Specifically, Zimmer and Engelkamp (1985) showed that memory of action phrases was worse if subjects had to perform a secondary motor task (e.g., a body related action as scratching oneself) compared to when only the action phrase had to be performed, which highlights the importance of the motor system (see also Ianì and Bucciarelli, 2017).

In contrast, the episodic integration theory suggests that there is no signal of the motor system, and the processing of action memory would mainly rely on conceptual processing (Zimmer et al., 2001). Studies supported this view by showing that enactment can improved the semantic integration (Kormi-nouri, 1995; Kormi-Nouri and Nilsson, 1998; Kormi-Nouri et al., 2003; Badinlou et al., 2018a, b). However, in our study, we found that SPT did not improve with semantic repetition. Thus, as enactment effect did not seem to benefit from conceptual processing, the present study does not support the episodic integration theory.

From the perspective of whether the enactment effect is associated with language representation, we examined the role of language and semantic repetition on the enactment effect to explore the degree of overlap and interaction between these factors. For the aforementioned result, remember that while the SPT condition improved memory, it was not affected by semantic repetition nor by language. Our viewpoint is in line with the idea that SPT is insensitive to cognitive strategies (e.g., Engelkamp and Zimmer, 1985; Ianì and Bucciarelli, 2018), thus partly pushing forward the notion that maybe the motor encoding is the additional factor that is fundamental to the enactment effect. However, it is possible that because SPT itself can be regarded as a deep encoding (e.g., Zimmer et al., 2001; Senkfor et al., 2008; Feyereisen, 2009), it may be repetition strategy in itself. It is therefore possible that the rehearsal used in the present study was not so effective to add something to SPT.^1^

Interestingly, language did not significantly affect the size of the enactment effect, indicating that enhanced performance in the SPT condition did not result from higher novelty or distinctiveness of English phrases but from the action itself. This is further in line with previous studies who investigated the enactment effect in more detail. They assessed participants’ memory of action phrases that participants did not enact themselves, but that they simply observed being performed by an experimenter (a condition labeled “experimenter-performed task,” EPT). Previous studies find that EPT improves memory compared to VT by facilitating relational processing, whereas SPT did not facilitate such relational processing (e.g., Von-Essen, 2005; Feyereisen, 2009). From the perspective of language representation, the current study provides further evidence that deploying the motor system specifically bolsters the salience of the actions by item-specific processing rather than by relational processing (see also Feyereisen, 2009). Nevertheless, it seems important to highlight that this is the first study to examine how language and semantic repetition interact with enacting action phrases. Although this gives novel insights in the mechanisms that contribute to the enactment effect, our conclusion remain somewhat preliminary at this point. Future studies will have to replicate the present work and will have to further disentangle the different factors and mechanisms that contribute to the enactment effect.

Finally, we carried out statistical analyses on the interactions between encoding type and language, separately for semantic repetition versus non-repetition. The results showed a significant interaction in the SPT condition for non-repeated phrases so that memory scores for English phrases were better than those of Chinese phrases. Showing that enacting can enhance foreign language learning. For future studies, it would be interesting to understand whether this effect would also persist when the foreign language is other than English. Overall, the fact that we found that actions can facilitate foreign language learning, places the current study in line with a recent study by Lajevardi et al. (2017), where the authors showed that the enactment of phrases was an effective encoding tool to learn a non-native language (see also Macedonia and von Kriegstein, 2012).

In sum, the present study demonstrated that the enactment effect is sufficiently robust to emerge in both native and non-native language (Chinese and English, respectively). More interestingly, the size of the effect was not significantly larger for phrases learned in the non-native language. While on one hand, we associate the overall enactment effect to the multimodal theory, on the other hand, we propose that superior memory performance for the foreign language is the result of “novelty item” effect. Regarding learning strategies, we found that conceptual elaboration does not improve SPT scores, however, VT scores improved with repeated semantics. Further, semantic repetition modulated the size of the enactment effect. We suggest that motor provides further item-specific information sufficient to memorize the items, partly in line with the multimodal theory.

## Data Availability Statement

The datasets generated for this study are available on request to the corresponding author.

## Ethics Statement

Ethical review and approval was not required for the study on human participants in accordance with the local legislation and institutional requirements. The patients/participants provided their written informed consent to participate in this study.

## Author Contributions

All authors listed have made a substantial, direct and intellectual contribution to the work, and approved it for publication.

## Conflict of Interest

The authors declare that the research was conducted in the absence of any commercial or financial relationships that could be construed as a potential conflict of interest.

## Footnotes

1 We thank a reviewer for pointing out this issue.

## References

[B1] Anderson D. R. Sweeney D. J. Williams T. A. (2011). *Statistics for Business and Economics.* Mason, IA: South-Western Cengage Learning.

[B2] Bäckman L. Nilsson L. G. Chalom D. (1986). New evidence on the nature of the encoding of action events. *Mem. Cogn.* 14 339–346. 10.3758/bf03202512 3762388

[B3] Badinlou F. Kormi-Nouri R. Knopf M. (2018a). Action memory and knowledge-based cuing in school-aged children: the effect of object presentation and semantic integration. *Acta Psychol.* 186 118–125. 10.1016/j.actpsy.2018.04.011 29705084

[B4] Badinlou F. Kormi-Nouri R. Knopf M. (2018b). A study of retrieval processes in action memory for school-aged children: the impact of recall period and difficulty on action memory. *J. Cogn. Psychol.* 30 792–802. 10.1080/20445911.2018.1535495

[B5] Badinlou F. Kormi-Nouri R. Mousavi Nasab S. H. Knopf M. (2017). Developmental differences in episodic memory across school ages: evidence from enacted events performed by self and others. *Memory* 25 84–94. 10.1080/09658211.2015.1126607 26711845

[B6] Borg C. Bouazza Z. Godeau M. Getenet J. C. Chainay H. (2018). Effect of emotion and type of encoding on memory for actions: verbal and subject-performed tasks. *Dement. Geriatr. Cogn. Disord.* 45 162–179. 10.1159/000488103 29843134

[B7] Cohen R. L. (1981). On the generality of some memory laws. *Scand. J. Psychol.* 22 267–281. 10.1111/j.1467-9450.1981.tb00402.x

[B8] Cohen R. L. (1989). Memory for action events: the power of enactment. *Educ. Psychol. Rev.* 1 57–80. 10.1007/bf01326550

[B9] Cohen R. L. Bryant S. (1991). The role of duration in memory and metamemory of enacted instructions (SPTs). *Psychol. Res.* 53 183–187. 10.1007/bf00941385

[B10] Cutica I. Ianì F. Bucciarelli M. (2014). Learning from text benefits from enactment. *Mem. Cogn.* 42 1026–1037. 10.3758/s13421-014-0417-y 24825120

[B11] Einstein G. O. McDaniel M. A. (1987). “Distinctiveness and the mnemonic benefits of bizarre imagery,” in *Imagery and Related Mnemonic Processes: Theories, Individual Difference, and Applications*, eds McDaniel M. A. Pressley M. (New York, NY: Springer Verlag), 78–102. 10.1007/978-1-4612-4676-3_4

[B12] Engelkamp J. (1996). Organisation and recall in verbal tasks and in subject-performed tasks. *Eur. J. Cogn. Psychol.* 8 257–274. 10.1080/095414496383086

[B13] Engelkamp J. (1998). *Memory for Actions.* Hove: Psychology Press.

[B14] Engelkamp J. (2001). “Action memory: a system-oriented approach,” in *Memory for Action: A Distinct form of Episodic Memory?*, eds Zimmer H. D. Cohen J. D. Guynn M. S. Engekalmp J. Kormi-Nouri R. Foley M. A. (New York, NY: Oxford University Press), 49–96.

[B15] Engelkamp J. Krumnacker H. (1980). Image- and motor-processes in the retention of verbal materials. *Z. Exp. Angew. Psychol.* 27 511–533.

[B16] Engelkamp J. Zimmer H. D. (1984). Motor programme information as a separable memory unit. *Psychol. Res.* 46 283–299. 10.1007/bf00308889 6494380

[B17] Engelkamp J. Zimmer H. D. (1985). Motor programs and their relation to semantic memory. *German J. Psychol.* 28 239–254.

[B18] Engelkamp J. Zimmer H. D. (1994). Motor similarity in subject-performed tasks. *Psychol. Res.* 57 47–53. 10.1007/bf00452995 7824684

[B19] Engelkamp J. Zimmer H. D. Mohr G. Sellen O. (1994). Memory of self-performed tasks: self-performing during recognition. *Mem. Cogn.* 22 34–39. 10.3758/bf03202759 8035683

[B20] Eysenck M. W. Keane M. T. (1995). *Cognitive Psychology: A Student’s Handbook*, 2nd Edn Hove: Erlbaum.

[B21] Feyereisen P. (2009). Enactment effects and integration processes in younger and older adults’ memory for actions. *Memory* 17 374–385. 10.1080/09658210902731851 19221926

[B22] Foley M. A. Ratner H. H. (2001). “The role of action-based structures in activity memory,” in *Memory for Action: A Distinct form of Episodic Memory?*, eds Zimmer H. D. Cohen R. L. Guynn M. J. Engelkamp J. Kormi-Nouri R. Foley M. A. (New York, NY: Oxford University Press), 112–135.

[B23] Gollyhäring C. Engelkamp J. (2003). Categorical-relational and order-relational information in memory for subject-performed and experimenter-performed actions. *J. Exp. Psychol. Learn. Mem. Cogn.* 29 965–975. 10.1037/0278-7393.29.5.965 14516228

[B24] Hainselin M. Picard L. Manolli P. Vankerkore-Candas S. Bourdin B. (2017). Hey teacher, don’t leave them kids alone: action is better for memory than reading. *Front. Psychol.* 8:325. 10.3389/fpsyg.2017.00325 28337159 PMC5343022

[B25] Hainselin M. Quinette P. Juskenaite A. Desgranges B. Martinaud O. de La Sayette V. (2014). Just do it! How performing an action enhances remembering in transient global amnesia. *Cortex* 50 192–199. 10.1016/j.cortex.2013.10.007 24268322

[B26] Helstrup T. (1987). One, two, or three memories? A problem-solving approach to memory for performed acts. *Acta Psychol.* 66 37–68. 10.1016/0001-6918(87)90017-5

[B27] Helstrup T. (1989). Loci for act recall: contextual influence on the processing of action events. *Psychol. Res.* 51 168–175. 10.1007/bf00309144 2616695

[B28] Helstrup T. (2005). In search of a motor element in memory for enacted events. *J. Cogn. Psychol.* 17 389–403. 10.1080/09541440440000087

[B29] Ianì F. Bucciarelli M. (2017). Mechanisms underlying the beneficial effect of a speaker’s gestures on the listener. *J. Mem. Lang.* 96 110–121. 10.1016/j.jml.2017.05.004

[B30] Ianì F. Bucciarelli M. (2018). Relevance of the listener’s motor system in recalling phrases enacted by the speaker. *Memory* 26 1084–1092. 10.1080/09658211.2018.1433214 29385905

[B31] Ianì F. Burin D. Salatino A. Pia L. Ricci R. Bucciarelli M. (2018). The beneficial effect of a speaker’s gestures on the listener’s memory for action phrases: the pivotal role of the listener’s premotor cortex. *Brain Lang.* 180–182 8–13. 10.1016/j.bandl.2018.03.001 29653280

[B32] Kausler D. H. Hakami M. K. (1983). Memory for topics of conversation: adult age differences and intentionality. *Exp. Aging Res.* 9 153–157. 10.1080/03610738308258444 6641774

[B33] Knopf M. Mack W. Lenel A. Ferrante S. (2005). Memory for action events: findings in neurological patients. *Scand. J. Psychol.* 46 11–19. 10.1111/j.1467-9450.2005.00430.x 15660629

[B34] Kormi-nouri R. (1995). The nature of memory for action events: an episodic integration view. *J. Cogn. Psychol.* 7 337–363. 10.1080/09541449508403103

[B35] Kormi-Nouri R. Moniri S. Nilsson L. G. (2003). Episodic and semantic memory in bilingual and monolingual children. *Scand. J. Psychol.* 44 47–54. 10.1111/1467-9450.00320 12603003

[B36] Kormi-Nouri R. Nilsson L. G. (1998). The role of integration in recognition failure and action memory. *Mem. Cogn.* 26 681–691. 10.3758/bf03211389 9701961

[B37] Kormi-Nouri R. Nilsson L. G. (2001). “The motor component is not crucial!” in *Memory for Action: A Distinct form of Episodic Memory*?, eds Zimmer H. D. Cohen R. Guynn M. J. Engelkamp J. Kormi-Nouri R. Foley M. A. (New York, NY: Oxford University), 97–111.

[B38] Krönke K. M. Mueller K. Friederici A. D. Obrig H. (2013). Learning by doing? The effect of gestures on implicit retrieval of newly acquired words. *Cortex* 49 2553–2568. 10.1016/j.cortex.2012.11.016 23357203

[B39] Kubik V. Obermeyer S. Meier J. Knopf M. (2014a). The enactment effect in a multi-trial free-recall paradigm. *J. Cogn. Psychol.* 26 781–787. 10.1080/20445911.2014.959018 21605121

[B40] Kubik V. Söderlund H. Nilsson L. G. Jönsson F. U. (2014b). Individual and combined effects of enactment and testing on memory for action phrases. *Exp. Psychol.* 61 347–355. 10.1027/1618-3169/a000254 24503878

[B41] Lajevardi N. Narang N. S. Marcus N. Ayres P. (2017). Can mimicking gestures facilitate learning from instructional animations and static graphics? *Comput. Educ.* 110 64–76. 10.1016/j.compedu.2017.03.010

[B42] Li G. Wang L. (2018). The role of item-specific information for the retrieval awareness of performed actions. *Front. Psychol.* 9:1325. 10.3389/fpsyg.2018.01325 30154741 PMC6102507

[B43] Li G. Wang L. Han Y. (2017). Directed forgetting of negative performed actions is difficult: a behavioral study. *Q. J. Exp. Psychol.* 70 53–61. 10.1080/17470218.2015.1120331 26624573

[B44] Liu S. Wang L. (2018). The association of motor information and verbal information: a new perspective on the mechanism of the SPT effect. *J. Cogn. Psychol.* 30 321–335. 10.1080/20445911.2018.1443463

[B45] Macedonia M. von Kriegstein K. (2012). Gestures enhance foreign language learning. *Biolinguistics* 6 393–416. 10.3389/fpsyg.2014.01467 25538671 PMC4260465

[B46] Masumoto K. Yamaguchi M. Sutani K. Tsuneto S. Fujita A. Tonoike M. (2006). Reactivation of physical motor information in the memory of action events. *Brain Res.* 1101 102–109. 10.1016/j.brainres.2006.05.033 16782071

[B47] Nilsson L.-G. (2000). “Remembering actions and words,” in *Oxford Handbook of Memory*, eds Craik F. I. M. Tulving E. (Oxford: Oxford University Press), 137–148.

[B48] Nilsson L.-G. Craik F. I. M. (1990). Additive and interactive effects in memory for subject-performed tasks. *Eur. J. Cogn. Psychol.* 2 305–324. 10.1080/09541449008406210

[B49] Nilsson L.-G. Kormi-Nouri R. (2001). “What is the meaning of a memory-systems approach? Comments on Engelkamp,” in *Memory for Action: A Distinct form of Episodic Memory?*, eds Zimmer H. D. Cohen R. Guynn M. J. Engelkamp J. Kormi-Nouri R. Foley M. A. (New York, NY: Oxford University), 136–143.

[B50] Russ M. O. Mack W. Grama C. R. Lanfermann H. Knopf M. (2003). Enactment effect in memory: evidence concerning the function of the supramarginal gyrus. *Exp. Brain Res.* 149 497–504. 10.1007/s00221-003-1398-4 12677330

[B51] Saltz E. Donnenwerth-Nolan S. (1981). Does motoric imagery facilitate memory for sentences? A selective interference test. *J. Verbal Learn. Verbal Behav.* 20 322–332. 10.1016/s0022-5371(81)90472-2

[B52] Schatz T. R. Spranger T. Knopf M. (2010). Is there a memory profit after repeated learning of subject-performed actions? Comparing direct and long-term memory performance level as a function of age. *Scand. J. Psychol.* 51 465–472. 10.1111/j.1467-9450.2010.00828.x 20546198

[B53] Schatz T. R. Spranger T. Kubik V. Knopf M. (2011). Exploring the enactment effect from an information processing view: what can we learn from serial position analyses? *Scand. J. Psychol.* 52 509–515. 10.1111/j.1467-9450.2011.00893.x 21605121

[B54] Schwartz R. N. Plass J. L. (2014). Click versus drag: user-performed tasks and the enactment effect in an interactive multimedia environment. *Comput. Hum. Behav.* 33 242–255. 10.1016/j.chb.2014.01.012

[B55] Seiler K. H. Engelkamp J. (2003). The role of item-specific information for the serial position curve in free recall. *J. Exp. Psychol. Learn. Mem. Cogn.* 29 954–964. 10.1037/0278-7393.29.5.954 14516227

[B56] Senkfor A. J. Van Petten C. Kutas M. (2008). Enactment versus conceptual encoding: equivalent item memory but different source memory. *Cortex* 44 649–664. 10.1016/j.cortex.2007.12.004 18472035 PMC2413056

[B57] Spranger T. Schatz T. R. Knopf M. (2008). Does action make you faster? A retrieval-based approach to investigating the origins of the enactment effect. *Scand. J. Psychol.* 49 487–495. 10.1111/j.1467-9450.2008.00675.x 18705671

[B58] Steffens M. C. Buchner A. Wender K. F. (2003). Quite ordinary retrieval cues may determine free recall of actions. *J. Mem. Lang.* 48 399–415. 10.1016/s0749-596x(02)00517-x

[B59] Steffens M. C. Jelenec P. Mecklenbräuker S. (2009). Decomposing the memory processes contributing to enactment effects by multinomial modelling. *Eur. J. Cogn. Psychol.* 21 61–83. 10.1080/09541440701868668

[B60] Steffens M. C. Jelenec P. Mecklenbräuker S. Marie Thompson E. (2006). Decomposing retrieval and integration in memory for actions: a multinomial modeling approach. *Q. J. Exp. Psychol.* 59 557–576. 10.1080/02724980443000764 16627356

[B61] Steffens M. C. von Stülpnagel R. Schult J. C. (2015). Memory recall after “learning by doing” and “learning by viewing”: boundary conditions of an enactment benefit. *Front. Psychol.* 6:1907. 10.3389/fpsyg.2015.01907 26733905 PMC4681778

[B62] Taleghani-Nikazm C. (2008). “Gestures in foreign language classrooms: an empirical analysis of their organization and function,” in *Selected Proceedings of the 2007 Second Language Research Forum*, eds Bowles M. Foote R. Perpiñán S. Bhatt R. (Somerville, MA: Cascadilla Proceedings Project), 229–238.

[B63] von Essen J. D. Nilsson L. G. (2003). Memory effects of motor activation in subject-performed tasks and sign language. *Psychon. Bull. Rev.* 10 445–449. 10.3758/bf03196504 12921422

[B64] Von-Essen J. D. (2005). Enactment enhances integration between verb and noun, but not relational processing, in episodic memory. *Scand. J. Psychol.* 46 315–321. 10.1111/j.1467-9450.2005.00461.x 16014075

[B65] Wammes J. D. Meade M. E. Fernandes M. A. (2017). Learning terms and definitions: drawing and the role of elaborative encoding. *Acta Psychol.* 179 104–113. 10.1016/j.actpsy.2017.07.008 28756291

[B66] Wippich W. Mecklenbräuker S. (1995). Implicit memory for textual materials. *Psychol. Res.* 57 131–141.

[B67] Yu Z. Wang L. (2017). Do physical properties affect enactment effect? The regulatory function of item familiarity. *Am. J. Psychol.* 130 315–327.

[B68] Zhao M. F. Zimmer H. D. Zhou X. Fu X. (2016). Enactment supports unitisation of action components and enhances the contribution of familiarity to associative recognition. *J. Cogn. Psychol.* 28 932–947. 10.1080/20445911.2016.1229321

[B69] Zimmer H. D. Cohen R. L. Guynn M. J. Engelkamp J. Korminouri R. Foley M. A. (2001). *Memory for Action. Memory for Action: A Distinct form of Episodic Memory?* New York, NY: Oxford University Press.

[B70] Zimmer H. D. Engelkamp J. (1985). An attempt to distinguish between kinematic and motor memory components. *Acta Psychol.* 58 81–106. 10.1016/0001-6918(85)90036-8 3976415

[B71] Zimmer H. D. Engelkamp J. (1999). Levels-of-processing effects in subject-performed tasks. *Mem. Cogn.* 27 907–914. 10.3758/bf03198543 10540819

